# Programmable N6-methyladenosine modification of *CDCP1* mRNA by RCas9-methyltransferase like 3 conjugates promotes bladder cancer development

**DOI:** 10.1186/s12943-020-01289-0

**Published:** 2020-12-03

**Authors:** Xiaoling Ying, Xu Jiang, Haiqing Zhang, Bixia Liu, Yapeng Huang, Xiaowei Zhu, Defeng Qi, Gang Yuan, Junhang Luo, Weidong Ji

**Affiliations:** 1 Center for Translational Medicine, The First Affiliated Hospital, Sun Yat-sen University, Guangzhou, 510080 China; 2 Department of Urology, The First Affiliated Hospital, Sun Yat-sen University, Guangzhou, 510080 China; 3 Department of Urology, Minimally Invasive Surgery Center, The First Affiliated Hospital of Guangzhou Medical University, Guangdong Key Laboratory of Urology, Guangzhou, 510230 China; 4 Department of Geratology, The First Affiliated Hospital, Sun Yat-sen University, Guangzhou, 510080 China

## Abstract

Accumulating evidence has revealed significant roles for N6-methyladenosine (m 6 A) modification in the development of various cancers. We previously demonstrated an oncogenic role of m 6 A-modified CUB domain containing protein 1 (CDCP1) in bladder cancer (BC) progression. However, the biological functions and underlying molecular mechanisms of engineered programmable m 6 A modification of CDCP1 mRNA in BC remain obscure. Here, we established a targeted m 6 A RNA methylation system by fusing the catalytic domain of methyltransferase like 3 (METTL3CD) to RCas9 as the RNA-targeting module. The constructed RCas9- METTL3 retained methylation activity and mediated efficient site-specific m 6 A installation in the presence of a cognate single guide RNA and short protospacer adjacent motif-containing ssDNA molecule . Subsequently, targeting m 6 A installation onto the 3′ untranslated region of CDCP1 promoted CDCP1 mRNA translation and facilitated BC development in vitro and in vivo. Our findings demonstrate that the RCas9-METTL3 system mediates efficient sitespecific m 6 A installation on CDCP1 mRNA and promotes BC development. Thus, the RCas9-METTL3 system provides a new tool for studying m 6 A function and a potential strategy for BC epitranscriptome-modulating therapies.

**Supplementary Information:**

**Supplementary information** accompanies this paper at 10.1186/s12943-020-01289-0.

## Background

RNA epitranscriptomics has gained popularity in recent years [1]. To date, more than 160 different RNA modifications have been identified [2]. Of these, N6-methyladenosine (m^6^A) is the most prevalent RNA modification in eukaryotes [3]. m^6^A modification is especially relevant to the occurrence and development of tumors. m^6^A methyltransferases may play oncogenic or suppressive roles in malignant tumors. Methyltransferase like 3 (METTL3) promotes the progression of bladder cancer (BC) by regulating the expression levels of *AFF4*, *IKBKB*, *RELA*, *MYC*, *ITGA6*, and CUB domain-containing protein 1 (*CDCP1*) or by accelerating pre-miR221/222 maturation in an m^6^A-dependent manner [4–7]. However, METTL14 inhibits the self-renewal capacity of BC-initiating cells and bladder tumorigenesis by modulating Notch1 m^6^A levels [8]. The methyltransferase family proteins recognize their specific sites and modify targeted transcripts differentially, suggesting that the location of the m^6^A modifications on mRNA transcripts may underlie the observed differences. As knockdown or overexpression of m^6^A methyltransferases leads to altered m^6^A content at numerous sites on many transcripts, it is difficult to determine the roles of specific m^6^A sites and reveal the causal relationships between individual m^6^A modifications and biological function. Therefore, generating an efficient, manipulative, and targeted site-specific m^6^A installation system will provide a critical tool for better understanding the role of locus-specific m^6^A modification in multiple biological processes.

Recent studies have repurposed CRISPR/Cas9 for RNA targeting (RCas9) by providing protospacer adjacent motif (PAM) as part of an oligonucleotide (PAMmer) that hybridizes to the target RNA. In our previous study, we reported an oncogenic role of m^6^A-modified *CDCP1* in BC progression. We hypothesized that the fusion of m^6^A regulators to RCas9 should manipulate m^6^A modification of *CDCP1* for exploring the biological function of locus-specific m^6^A RNA methylation.

In this study, we linked the METTL3 catalytic domain (METTL3CD) to the N-terminus of dCas9, fused two SV40 nuclear localization signal (NLS) sequences at the C terminus, and established a targeted RNA methylation system that enables site-directed m^6^A incorporation in target transcripts by targeting single guide (sg) RNAs and short PAM-containing ssDNA molecules (PAMmers) against *CDCP1*. We demonstrate that targeting m^6^A installation onto the 3′ untranslated region (UTR) of *CDCP1* enhances *CDCP1* mRNA translation and facilitates BC development in vitro and in vivo.

## Results and discussions

### The RCas9-METTL3 system enhances m^6^A modification

**Fig. 1:**
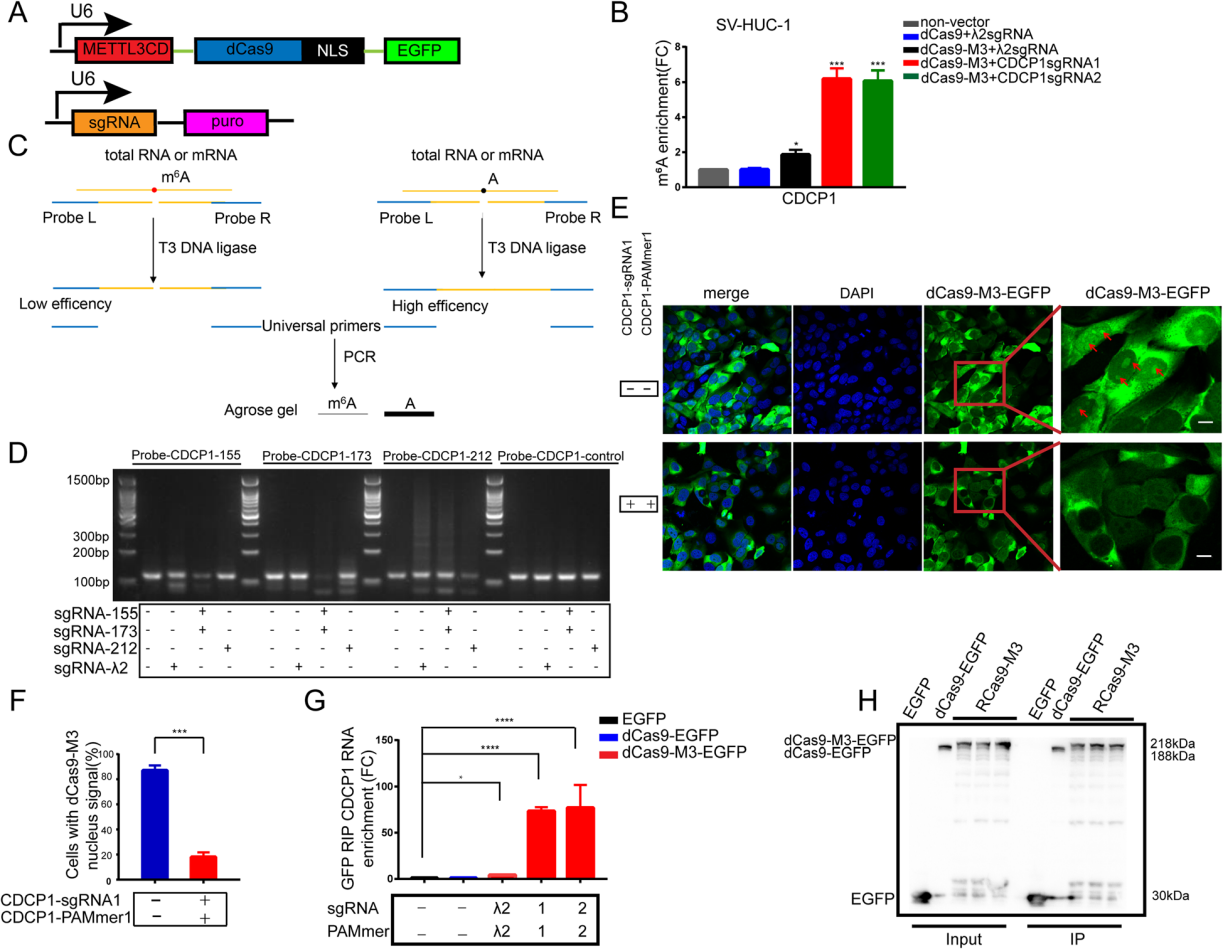
RCas9-methyltransferase like 3 (METTL3) system enhances N6-methyladenosine (m^6^A) modification. **a** Upper panel: a schematic representation of the deactivated Cas9 (dCas9) and METTL3 catalytic domain (METTL3CD) fusion proteins. Lower panel: a modified single guide (sg) RNA construct with puromycin. **b** Methylated RNA immunoprecipitation (MeRIP) assays showing the abundance of m^6^A of CUB domain-containing protein 1 **(***CDCP1*) mRNA in SV-HUC-1 cells expressing RCas9-M3. **c** A schematic diagram of the ligation-amplification method for single-base m^6^A validation. **d** Validation results of four different sites. The positions of the m^6^A sites (155, 173, and 212) were numbered relative to the first nucleotide of the 3′ untranslated region (UTR). The three sites were confirmed as m^6^A sites, whereas the other site was unmodified and treated as a control. **e** dCas9-M3 nuclear co-export with *CDCP1* mRNA. The dCas9-M3 was delivered to SV-HUC-1 cells with a sgRNA and short protospacer adjacent motif-containing ssDNA molecule (PAMmer) targeting the 3′ UTR of *CDCP1*. Scale bars: 10 μm. Blue pseudocolor: 4′,6-diamidino-2-phenylindole staining of the nuclei. **f** Fraction of cells with a nuclear signal. Mean values ± standard error of the mean (SEM; *n* = 40). **g**, **h** RIP assays of enhanced green fluorescent protein (EGFP) after stable transfection of the RCas9-M3 system in SV-HUC-1 cells targeting *CDCP1* mRNA compared to non-targeting sgRNA and PAMmer or EGFP alone. **g** Real-time quantitative polymerase chain reaction (qRT-PCR) analysis of RCas9-M3 RIP in SV-HUC-1 cells with *CDCP1* 3′ UTR primers. **h**. Western blot of EGFP. Data are presented as the mean ± SEM (*n* = 3). *p < 0.05, **p < 0.01, ***p < 0.001, and ****p < 0.0001

To establish a targeted RNA methylation system, we fused the METTL3CD to the N-terminus of nuclease-null Cas9 (dCas9) tagged with an enhanced green fluorescent protein (EGFP; Fig. 1a). Then, dCas9 was fused to two SV40 NLS sequences at the C terminus. Primer sequences for polymerase chain reaction (PCR) amplification are summarized in Table S1. Next, we sought to test specific m^6^A levels in human cells. We designed guide RNAs targeting the 3′ UTR of *CDCP1* or epidermal growth factor receptor *(EGFR)* mRNAs. We designed a λ2 sgRNA-PAMmer pair as a negative control (sgRNA and PAMmer sequences are listed in Table S2). The methylated RNA immunoprecipitation (MeRIP) assays and real-time quantitative PCR (RT-qPCR; the primers used are listed in Table S4) of *CDCP1* revealed six- to seven-fold higher methylation from *CDCP1*-targeted dCas9–M3 but none from dCas9 without methyltransferase constructs (Fig. 1b). We observed a smaller increase (1.8-fold) in m^6^A modification from dCas9–M3 when combined with λ2 guide RNA, suggesting potential off-target methylation by this construct. Moreover, we confirmed these results by targeting an *EGFR* transcript. In agreement with the *CDCP1* results, RCas9-M3 increased m^6^A levels of *EGFR* (60.5-, 32.7-, and 24.6-fold, respectively) only when fused to dCas9-M3 and supplied with *EGFR*-targeting guide RNA-PAMmers (Fig. S1). To further confirm the effect of METTL3CD-RCas9 on targeting site-specific m^6^A modifications, we designed probe L and probe R against the *CDCP1* 3′ UTR at three m^6^A sites (155, 173, and 212; numbered relative to the first nucleotide of the 3′ UTR) (Table S3) [7] and used the T3 ligase to concatenate the two probes onto templates that could be amplified by PCR. Thus, the amount of PCR products could be used to assess ligation efficiency and indicate the methylation status of each site (Fig. 1c). In transfected SV-HUC-1 cells, sgRNAs (155, 173, and 212) increased methylation at the #155, #173, and #212 sites, respectively (Fig. 1d), indicating that our constructed RCas9-M3 system can mediate efficient site-specific m^6^A modification. Our system-established stable cell lines via lentiviral transduction, providing an ideal tool for dissecting the biological function of locus-specific m^6^A RNA methylation.

Next, we assessed if RCas9-M3 could recognize specific mRNA substrates in human cells by testing whether dCas9-M3 containing an NLS tagged with EGFP and mRNA was co-exported from the nucleus in the presence of a cognate sgRNA and PAMmer designed to recognize the mRNA. We transfected dCas9-M3 into SV-HUC-1 cells and observed that 87% of cells showed an EGFP signal in the nucleus. When the cells were co-transfected with CDCP1-targeting sgRNA plasmid and PAMmer, only 18% of cells showed an EGFP signal in the nucleus (Fig. 1e and f). These observations suggest that dCas9-M3 is exported from the nucleus in the presence of a cognate sgRNA and PAMmer, consistent with a previous finding that RCas9 is exported from the nucleus in the presence of sgRNA targeting glyceraldehyde 3-phosphate dehydrogenase mRNA [9]. To further confirm that RCas9-M3 was exported from the nucleus by binding to target mRNA, RIP experiments were performed. Western blot analysis revealed that the EGFP or EGFP fusion proteins were of the expected size (Fig. 1h and Fig. S2a). RT-qPCR revealed that the relative abundance of *CDCP1* or *EGFR* in targeting groups was significantly higher than that in non-targeting groups (Fig. 1g and Fig. S2b), indicating that the RCas9-M3 system binds to targeted mRNAs and mediates efficient site-specific m^6^A installation.

To evaluate the off-target effects of the RCas9-M3 system, we first predicted off-target gRNA binding sites for *CDCP1* gRNAs by BLASTN using “somewhat similar sequences.” We chose the top three matching genes for sgRNA1/2 of *CDCP1* (Fig. S3a). The methylation level of these off-target sites was detected by MeRIP-qPCR after transfection with dCas9–M3 and *CDCP1* targeting sgRNAs or a non-targeting λ2 sgRNA. The results showed no significant effect on the methylation levels of these off-target loci (Fig.S3b), although two of them (SPECC1L, OR4A5) had slightly increased methylation, indicating that the off-target effect on tested transcripts was limited. Furthermore, we performed differential RNA-seq analysis to examine the effect of RCas9-M3 on the transcriptome. The results showed that a comparison of these constructs with non-targeting λ2 sgRNA control revealed more (> 1400 out of > 14,730 total genes analyzed) differentially expressed genes (false discovery rate (FDR) corrected *P* < 0.05 and more than twofold change; see Fig. S3c). Those changes in transcription may be caused by target methylation from RCas9-M3 upregulating CDCP1 protein levels. However, there were only a few (< 240 of > 14,730 total genes analyzed) differentially expressed genes between the CDCP1-sgRNA155–173 and CDCP1-sgRNA212 groups (Fig. S3d). These differences may be due to different m^6^A methylation levels in the two sgRNA groups. These findings suggest that RCas9-M3 exhibits satisfactory on-target efficiency.

## Targeted methylation using RCas9-METTL3 promotes translation

We previously showed that m^6^A promotes the translation of *CDCP1* mRNA and promotes bladder tumor growth [7]. m^6^A promotes lung cancer cell growth, survival, and invasion by enhancing *EGFR* mRNA translation [10]. To investigate whether RCas9-M3 facilitates the translation of targeted mRNAs, we performed a dual-luciferase reporter assay in 293 T using dual-luciferase vectors (psiCHECK-2) with a segment of the CDCP1 3′ UTR containing m^6^A sites or mutation of the three motifs (Fig. S4a). The results showed that the RCas9-M3 system significantly increased wild-type luciferase activity but not m^6^A motif-mutated luciferase activity compared to non-target control (Fig. S4b). We confirmed these results in stable SV-HUC-1 cells containing RCas9-M3. The wild-type or mutant-type psiCHECK-2-*CDCP1*–3′ UTR was transfected into the stable cells, and the relative fluorescence signal was measured. The results revealed that the wild-type but not mutant-type luciferase activity in the target group was significantly higher than that in the non-target groups (Fig. S4c). Even with a non-targeting PAMmer, cells containing dCas9-M3 using target sgRNA had higher luciferase activity than cells with non-target sgRNA. Luciferase activity was enhanced by the target PAMmer (Fig. S4b and S4c), suggesting that the sgRNA is the primary determinant of RNA substrate recognition. These results are consistent with the previous programmable RNA tracking in live cells showing that RNA binding by Cas9:sgRNA is independent of, but strengthened by, PAMmer [9]. Furthermore, *CDCP1*-sgRNA2 showed higher luciferase activity than *CDCP1*-sgRNA1 in stable SV-HUC-1 cells (Fig. S4c), suggesting that *CDCP1*-sgRNA2 has a higher translation effect. Next, we evaluated protein expression from the target genes using western blot analysis. *CDCP1* or *EGFR* protein levels were significantly elevated in stably transfected SV-HUC-1 or METTL3-depleted HeLa cells compared to control cells (Fig. S4e and S4f). However, there was no significant difference in mRNA expression among each group (Fig. S4e and S4f). An immunofluorescence assay was performed to further confirm the western blot results (Fig. S4h). These results suggest that the RCas9-M3 targeted modification system can upregulate the translation of target genes.

## Targeting of the *CDCP1* m^6^A by RCas9-M3 facilitates BC development

**Fig. 2:**
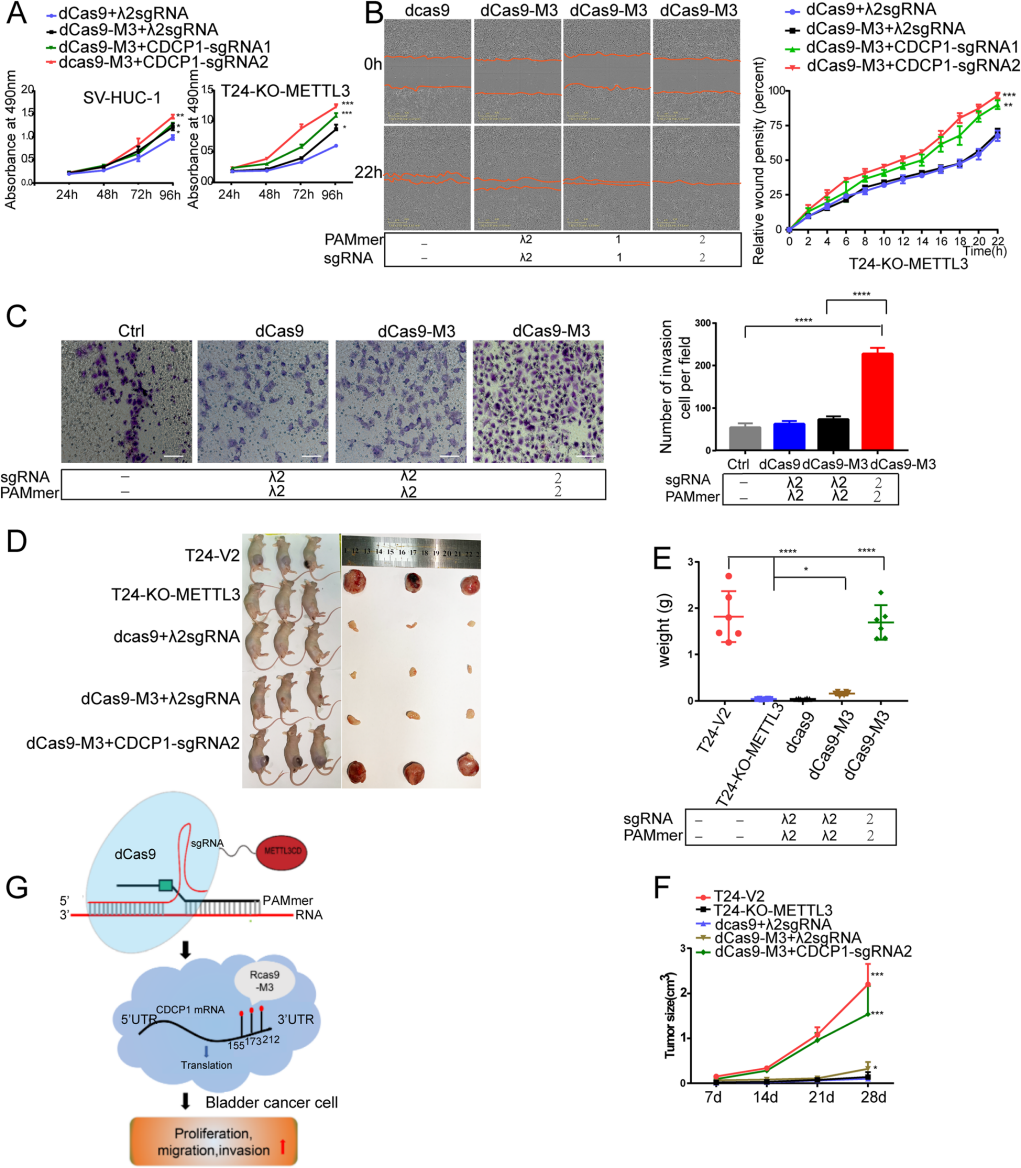
Targeting of the *CDCP1* m^6^A by RCas9-M3 facilitates bladder cancer (BC) development. **a-c** Targeted methylation by the RCas9-M3 system facilitates cancer progression in vitro. **a** MTT assay of cellular proliferation in SV-HUC-1 and METTL3-depleted T24 cells expressing the RCas9-M3 system. **b** RCas9-M3 targeting of *CDCP1* mRNA promotes cell migration. Scale bars: 100 μm; 200×. **c** RCas9-M3 targeting of *CDCP1* mRNA promotes cell invasion. Scale bars: 100 μm; 200×. **d-f** Targeted methylation by the RCas9-M3 system facilitates cancer progression in vivo. Representative tumor images **d**, weights **e**, and growth curves **f** are shown (*n* = 6). *p < 0.05, **p < 0.01, ***p < 0.001, and ****p < 0.0001

To ascertain the function of the RCas9-M3 system in BC development, RCas9-M3 or control plasmids were stably transfected into SV-HUC-1 or METTL3-depleted HeLa cells. The MTT assay results showed that the cell proliferation rate in the RCas9-M3 targeting group was significantly higher than that in the non-targeting control group (Fig. 2a). Cell migration and invasion were enhanced in RCas9-M3 targeting cells compared to control cells (Fig. 2b and c). Furthermore, the *CDCP1*-sgRNA2 targeting group in METTL3-depleted T24 cells had higher viability and migration than the *CDCP1*-sgRNA1 targeting group and the non-targeting groups (Fig. 2a and b). Moreover, *CDCP1*-sgRNA2 showed higher luciferase activity than *CDCP1*-sgRNA1 in stable SV-HUC-1 cells (Fig. S4c). To further determine whether RCas9-M3 also facilitates BC development in vivo, METTL3-depleted T24 cells were stably transfected with dCas9, dCas9-M3, *CDCP1*-sgRNA2, *CDCP1*-PAMmer2, or λ2 sgRNA-PAMmer, and the transfected cells were injected into nude mice (5 × 10^6^ cells per mouse). After 4 weeks, the tumors were dissected from the mice and weighed (Fig. 2d and e). The tumor growth curves were drawn according to tumor volume and implantation timepoint (Fig. 2f). Knockout of METTL3 remarkably reduced tumor volume and weight. However, tumors arising from METTL3-depleted T24 cells containing dCas9-M3 and *CDCP1*-sgRNA2-PAMmer2 had significantly higher volume and weight than the non-targeting groups (Fig. 2e and f). These in vitro and in vivo results indicate that the RCas9-M3 system promotes BC progression.

## Conclusions

Our constructed RCas9-M3 system can achieve targeted modification of mRNA and promote BC development. Thus, the RCas9-M3 system via lentiviral transduction is a powerful tool for exploring the biological effect of locus-specific m^6^A RNA methylation and presents a novel strategy for targeted interventions in BC or RNA modification defect-related diseases.

## Supplementary Information

**Additional file 1: Supplementary Figure 1**. MeRIP and RT-qPCR of epidermal growth factor receptor (*EGFR*) targeted by RCas9-M3 editors. (a) Western blot analysis of *EGFR* expression in control and METTL3-depleted cells (HeLa-KO-M3). (b) m^6^A enrichment of the *EGFR* mRNA 3′ UTR in METTL3-depleted cells (HeLa-KO-M3) with the RCas9 system. All qRT-PCR data are presented as the mean ± SEM (*n* = 3). *p < 0.05 and ****p < 0.0001. **Supplementary Figure 2.** The RCas9-M3 system binds to targeted mRNA. (a, b) RIP assays of EGFP after transfection of the *EGFR* mRNA-targeting RCas9-M3 system into METTL3-depleted HeLa cells compared to non-targeting sgRNA and PAMmer or EGFP alone. (a) Western blots of EGFP proteins. (b) qRT-PCR analysis of RCas9-M3 RIP in METTL3-depleted HeLa cells with *EGFR* 3′ UTR primers. Data are presented as the mean ± SEM (n = 3). *p < 0.05, ****p < 0.0001. **Supplementary Figure 3**. Effects of off-target methylation of RCas9-M3 and RCas9-M3 on cellular transcriptome abundances. (a) Sequence alignment between *CDCP1*-sgRNAs targeting sequences and SPECC1L, AP4S1, OR4A5, SLC22A9, ST18 or ROR1 mRNAs. (b) SV-HUC-1 Cells were stably transfected with dCas9-METTL3 and λ2-gRNA or *CDCP1*-sgRNA155–173/212, with m^6^A levels of SPECC1L, AP4S1, OR4A5, SLC22A9, ST18 or ROR1 measured by m^6^A -RIP-qPCR analysis. (c, d) Volcano plots depicting differential gene transcript abundance in SV-HUC-1 cells transfected with (c) *CDCP1*-sgRNA155–173 compared to λ2 guide RNA and (d) *CDCP1*-sgRNA155–173 compared to *CDCP1*-sgRNA212. The cells were co-transfected with RCas9-M3 for all conditions. Differentially expressed genes (*p* < 0.05 and fold-change > 2) are shown in red and counted in each volcano plot. Over 14,730 total genes were analyzed in each experiment. Statistical significance was calculated using a two-tailed Student’s *t*-test with a false discovery rate correction. Data are presented as mean ± SEM from three independent experiments. NS, no significant. **Supplementary Figure 4.** Targeted methylation using RCas9-METTL3 promotes translation**.** (a) A psiCHECK-2 luciferase reporter plasmid carrying a fragment of the human *CDCP1* 3′ UTR containing three putative m^6^A motifs (target sites for RCas9). The positions of the m^6^A sites (155, 173, and 212) were numbered relative to the first nucleotide of the 3′ UTR. The sgRNAs were designed to target the three putative m^6^A motifs. Relative luciferase activity reveals the translational effects of RCas9-M3 binding to mRNA. (b) Relative luciferase activity of psiCHECK-2-*CDCP1*–3′ UTR after transient transfection of the RCas9-M3 system in HEK293T cells targeting the *CDCP1* mRNA compared to non-targeting control. (c) Relative luciferase activity of the psiCHECK-2-*CDCP1* 3′ UTR after stable transfection of the *CDCP1* mRNA-targeting RCas9-M3 system in SV-HUC-1 cells compared to non-targeting control. (d) qRT-PCR analysis of *CDCP1* mRNA levels in control and SV-HUC-1 cells expressing the RCas9 system. mRNA was normalized to β-Actin mRNA. The relative ratio (fold-change) obtained from SV-HUC-1 cells without RCas9-M3 was set to 1. (e) Western blot analysis of *CDCP1* expression in control (SV-HUC-1 without RCas9 and RCas9 without fused METTL3) and stably transfected SV-HUC-1 cells expressing the RCas9 system. (f) Western blot analysis of *EGFR* expression in control (METTL3-depleted HeLa cells without RCas9-M3, RCas9 without fused METTL3CD, or EGFP alone) and METTL3-depleted HeLa cells transfected with the RCas9 system. (g) qRT-PCR analysis of *EGFR* mRNA levels. (h) Protein expression of *CDCP1* in stable SV-HUC-1 cells expressing the RCas9-M3 system. All bar plot data are presented as the mean ± SEM of three independent experiments. *p < 0.05, **p < 0.01, and ***p < 0.001. **Supplementary Table 1.** PCR primer sequences. **Supplementary Table 2.** sgRNA and PAMer sequences. **Supplementary Table 3**. Ligase-based probes and primer sequences. **Supplementary Table 4.** RT-qPCR primer sequences.

## Data Availability

All data generated or analyzed during this study are included in this manuscript (and its supplementary information files).

## Abbreviations

BC: Bladder cancer
m^6^A: N6-methyladenosine
METTL3: Methyltransferase like 3
MeRIP: Methylated RNA immunoprecipitation
RT-qPCR: Real-time quantitative polymerase chain reaction
CDCP1: CUB domain-containing protein 1
EGFR: Epidermal growth factor receptor
EGFP: Enhanced green fluorescent protein
PAMmer: Protospacer adjacent motif
NLS: Nuclear localization signal
