# Supplementary material for: Programmable N6-methyladenosine modification of CDCP1 mRNA by RCas9-methyltransferase like 3 conjugates promotes bladder cancer development
Source: Mol Cancer. 2020 Dec 3;19:169. doi: 10.1186/s12943-020-01289-0 (PMC7712571; doi:10.1186/s12943-020-01289-0)
Supplement: Supplementary file 1 — Additional file 1: Supplementary Figure 1. MeRIP and RT-qPCR of epidermal growth factor receptor (EGFR) targeted by RCas9-M3 editors. (a) Western blot analysis of EGFR expression in control and METTL3-depleted cells (HeLa-KO-M3). (b) m6A enrichment of the EGFR mRNA 3′ UTR in METTL3-depleted cells (HeLa-KO-M3) with the RCas9 system. All qRT-PCR data are presented as the mean ± SEM (n = 3). *p < 0.05 and ****p < 0.0001. Supplementary Figure 2. The RCas9-M3 system binds to targeted mRNA. (a, b) RIP assays of EGFP after transfection of the EGFR mRNA-targeting RCas9-M3 system into METTL3-depleted HeLa cells compared to non-targeting sgRNA and PAMmer or EGFP alone. (a) Western blots of EGFP proteins. (b) qRT-PCR analysis of RCas9-M3 RIP in METTL3-depleted HeLa cells with EGFR 3′ UTR primers. Data are presented as the mean ± SEM (n = 3). *p < 0.05, ****p < 0.0001. Supplementary Figure 3. Effects of off-target methylation of RCas9-M3 and RCas9-M3 on cellular transcriptome abundances. (a) Sequence alignment between CDCP1-sgRNAs targeting sequences and SPECC1L, AP4S1, OR4A5, SLC22A9, ST18 or ROR1 mRNAs. (b) SV-HUC-1 Cells were stably transfected with dCas9-METTL3 and λ2-gRNA or CDCP1-sgRNA155–173/212, with m6A levels of SPECC1L, AP4S1, OR4A5, SLC22A9, ST18 or ROR1 measured by m6A -RIP-qPCR analysis. (c, d) Volcano plots depicting differential gene transcript abundance in SV-HUC-1 cells transfected with (c) CDCP1-sgRNA155–173 compared to λ2 guide RNA and (d) CDCP1-sgRNA155–173 compared to CDCP1-sgRNA212. The cells were co-transfected with RCas9-M3 for all conditions. Differentially expressed genes (p < 0.05 and fold-change > 2) are shown in red and counted in each volcano plot. Over 14,730 total genes were analyzed in each experiment. Statistical significance was calculated using a two-tailed Student’s t-test with a false discovery rate correction. Data are presented as mean ± SEM from three independent experiments. NS, no significant. Supplementary Figure 4. Targeted methylat [file 12943_2020_1289_MOESM1_ESM.docx]

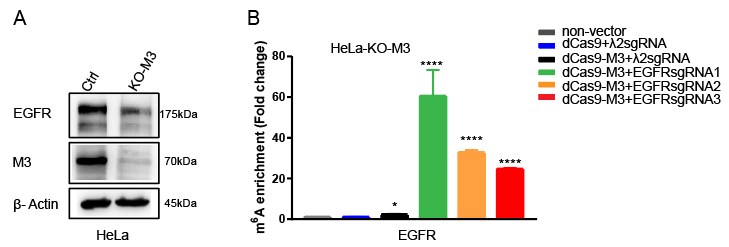


**Supplementary Fig. 1.** **MeRIP and RT-qPCR of epidermal growth factor receptor (*EGFR*) targeted by RCas9-M3 editors.** (a) Western blot analysis of *EGFR* expression in control and METTL3-depleted cells (HeLa-KO-M3). (b) m^6^A enrichment of the *EGFR* mRNA 3ʹ UTR in METTL3-depleted cells (HeLa-KO-M3) with the RCas9 system. All qRT-PCR data are presented as the mean ± SEM (n = 3). *p < 0.05 and ****p < 0.0001.


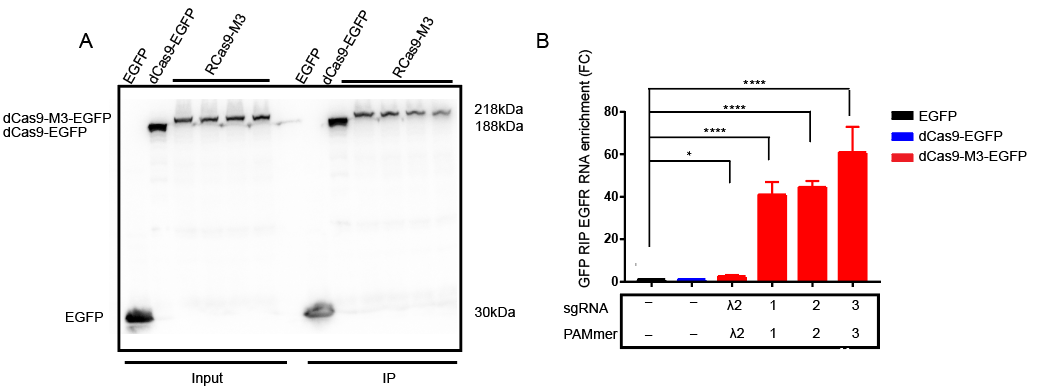


**Supplementary Fig. 2. The RCas9-M3 system binds to targeted mRNA.** (a, b) RIP assays of EGFP after transfection of the *EGFR* mRNA-targeting RCas9-M3 system into METTL3-depleted HeLa cells compared to non-targeting sgRNA and PAMmer or EGFP alone. (a) Western blots of EGFP proteins. (b) qRT-PCR analysis of RCas9-M3 RIP in METTL3-depleted HeLa cells with *EGFR* 3ʹ UTR primers. Data are presented as the mean ± SEM (n = 3). *p < 0.05, ****p < 0.0001.


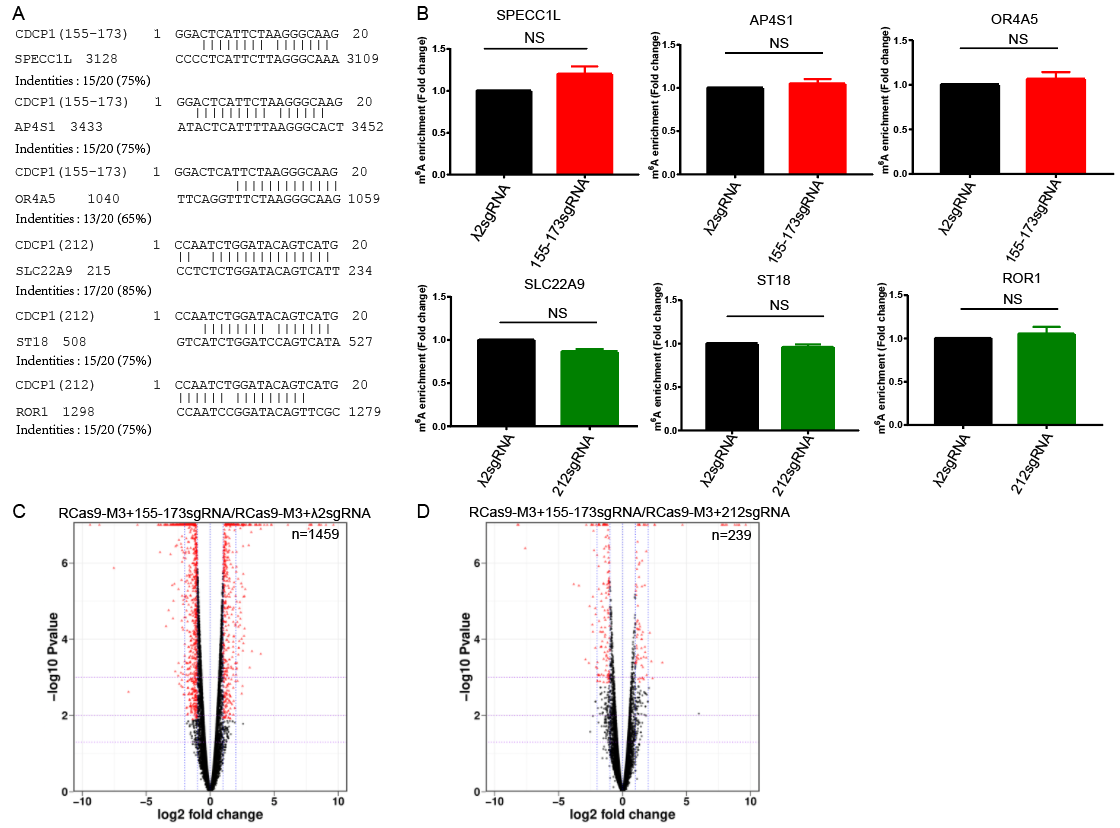


**Supplementary Fig. 3. Effects of off-target methylation of RCas9-M3 and RCas9-M3 on cellular transcriptome abundances.** (a) Sequence alignment between *CDCP1*-sgRNAs targeting sequences and SPECC1L, AP4S1, OR4A5, SLC22A9, ST18 or ROR1 mRNAs. (b) SV-HUC-1 Cells were stably transfected with dCas9-METTL3 and λ2-gRNA or *CDCP1*-sgRNA155-173/212, with m^6^A levels of SPECC1L, AP4S1, OR4A5, SLC22A9, ST18 or ROR1 measured by m^6^A -RIP-qPCR analysis. (c, d) Volcano plots depicting differential gene transcript abundance in SV-HUC-1 cells transfected with (c) *CDCP1*-sgRNA155-173 compared to λ2 guide RNA and (d) *CDCP1*-sgRNA155-173 compared to *CDCP1*-sgRNA212. The cells were co-transfected with RCas9-M3 for all conditions. Differentially expressed genes (p < 0.05 and fold-change > 2) are shown in red and counted in each volcano plot. Over 14,730 total genes were analyzed in each experiment. Statistical significance was calculated using a two-tailed Student’s *t*-test with a false discovery rate correction. Data are presented as mean ±SEM from three independent experiments. NS, no significant.


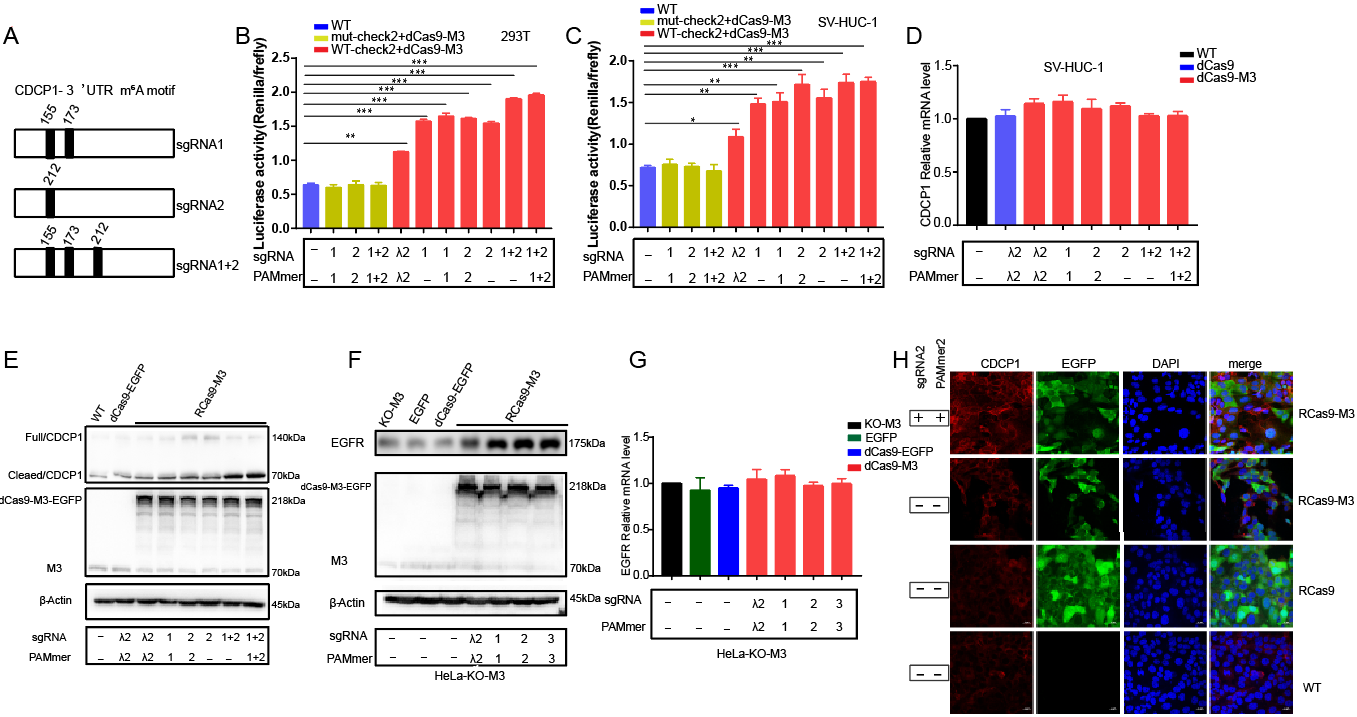


**Supplementary Fig. 4.**  **Targeted methylation using RCas9-METTL3 promotes translation.** (a) A psiCHECK-2 luciferase reporter plasmid carrying a fragment of the human *CDCP1* 3ʹ UTR containing three putative m^6^A motifs (target sites for RCas9). The positions of the m^6^A sites (155, 173, and 212) were numbered relative to the first nucleotide of the 3ʹ UTR. The sgRNAs were designed to target the three putative m^6^A motifs. Relative luciferase activity reveals the translational effects of RCas9-M3 binding to mRNA. (b) Relative luciferase activity of psiCHECK-2-*CDCP1*-3ʹ UTR after transient transfection of the RCas9-M3 system in HEK293T cells targeting the *CDCP1* mRNA compared to non-targeting control. (c) Relative luciferase activity of the psiCHECK-2-*CDCP1* 3ʹ UTR after stable transfection of the *CDCP1* mRNA-targeting RCas9-M3 system in SV-HUC-1 cells compared to non-targeting control. (d) qRT-PCR analysis of *CDCP1* mRNA levels in control and SV-HUC-1 cells expressing the RCas9 system. mRNA was normalized to β-Actin mRNA. The relative ratio (fold-change) obtained from SV-HUC-1 cells without RCas9-M3 was set to 1. (e) Western blot analysis of *CDCP1* expression in control (SV-HUC-1 without RCas9 and RCas9 without fused METTL3) and stably transfected SV-HUC-1 cells expressing the RCas9 system. (f) Western blot analysis of *EGFR* expression in control (METTL3-depleted HeLa cells without RCas9-M3, RCas9 without fused METTL3CD, or EGFP alone) and METTL3-depleted HeLa cells transfected with the RCas9 system. (g) qRT-PCR analysis of *EGFR* mRNA levels. (h) Protein expression of *CDCP1* in stable SV-HUC-1 cells expressing the RCas9-M3 system. All bar plot data are presented as the mean ± SEM of three independent experiments. *p < 0.05, **p < 0.01, and ***p < 0.001.

| 60954-dcas9-2×NLS-F | AAACCTAAGAATTTAATGGACAAGAAGTACAGCATCG |
| --- | --- |
| 60954-dcas9-2×NLS-R | GCCCTTGCTCACCATGGATCCGCTGCTGCCGTTGCTC |
| pcDNA3.1-dcas9-2×NLS-F | AAACCTAAGAATTTAATGGACAAGAAGTACAGCATCG |
| pcDNA3.1-dcas9-2×NLS-R | GCCCTTGCTCACCATGGATCCGCTGCTGCCGTTGCTC |
| pcDNA3.1-METTL3-F | TTGGTACCGAGCTCGCAGAGTGTCGGAGGTGATTCCA |
| pcDNA3.1-METTL3-R | GTACTTCTTGTCCATTAAATTCTTAGGTTTAGAGATG |
| pcDNA3.1-EGFP-F | GGCAGCAGCGGATCCATGGTGAGCAAGGGCGAGGAGC |
| pcDNA3.1-EGFP-R | GCGGGCCCTCTAGACTTACTTGTACAGCTCGTCCATG |
| 60954-METTL3-F | TCTCGAGAATTCTCACAGAGTGTCGGAGGTGATTCCA |
| 60954-METTL3-R | GTACTTCTTGTCCATTAAATTCTTAGGTTTAGAGATG |
| 60954-dcas9-F | TCTCGAGAATTCTCACGCGTATGGACAAGAAGTACAGCATCG |
| 60954-dcas9-R | GCCCTTGCTCACCATGGATCCGCTGCTGCCGTTGCTC |
| 60954-dcas9-EGFP-F | GGCAGCAGCGGATCCATGGTGAGCAAGGGCGAGGAGC |
| 60954-dcas9-EGFP-R | CGACTCTAGAGTCGCGGCCGCTTACTTGTACAGCTCGTCCATG |
| 60954-EGFP-F | GGCAGCAGCGGATCCATGGTGAGCAAGGGCGAGGAGC |
| 60954-EGFP-R | CGACTCTAGAGTCGCTTACTTGTACAGCTCGTCCATG |
| BsmBI-scaffold-cppt-F | CTTGTGGAAAGGACGAAA CACCGGAGACGGACGTCTCTGTT |
| BsmBI-scaffold-cppt-R | TTGCACCCGGGCCCCCTCGAGCCGGCGCCAAAGTGGATC |

**Supplementary Table 1.** PCR primer sequences

**Supplementary Table 2.** sgRNA and PAMer sequences

| CDCP1-sgRNA1-oligo1 | caccGCTTGCCCTTAGAATGAGTCC |
| --- | --- |
| CDCP1-sgRNA1-oligo2 | aaacGGACTCATTCTAAGGGCAAGC |
| CDCP1-sgRNA2-oligo1 | caccGCATGACTGTATCCAGATTGG |
| CDCP1-sgRNA2-oligo2 | aaacCCAATCTGGATACAGTCATGC |
| EGFR-sgRNA1-oligo1 | caccGCTGGGTATCGAAAGAGTCTG |
| EGFR-sgRNA1-oligo2 | aaac CAGACTCTTTCGATACCCAGC |
| EGFR-sgRNA2-oligo1 | caccGTGAAGACAAAGGAATGCAAC |
| EGFR-sgRNA2-oligo2 | aaac GTTGCATTCCTTTGTCTTCAC |
| EGFR-sgRNA3-oligo1 | caccGTAAGGAAGAATTCCATCCCC |
| EGFR-sgRNA3-oligo2 | aaac GGGGATGGAATTCTTCCTTAC |
| λ2-sgRNA-oligo1 | caccGTGATAAGTGGAATGCCATG |
| λ2-sgRNA-oligo2 | aaacCATGGCATTCCACTTATCAC |
| CDCP1-PAMer-1 | mATmGAmGTmCCmUGGmGAmGCmAAmUGmUGmAAmGTmUG |
| CDCP1-PAMer-2 | mCAmGAmUTmGGmUGGmUCmATmCAmUTmUTmCAmATmGT |
| EGFR-PAMer-1 | mAGmAGmUCmUGmUGGmUTmUTmAGmGGmCTmCAmUAmCT |
| EGFR-PAMer-2 | mAAmUGmCAmACmUGGmCCmAAmAAmUGmUGmCCmCGmAG |
| EGFR-PAMer-3 | mCCmATmCCmCCmUGGmGTmUTmCTmUCmUTmUGmCCmCA |
| λ2-PAMer | mATmGCmCAmUGmUGGmGCmUGmUCmAAmAAmUTmGAmGC |

**Supplementary Table 3**. Ligase-based probes and primer sequences

| CDCP1（155）Probe-L1 | PO_4_CCACTGAGCAATGTGAAGTTGGCGGTGTCCAGGAAAACCTCTCTATGGGCAGTCGGTGAT |
| --- | --- |
| CDCP1（155）Probe-R1 | CCATCTCATCCCTGCGTGTCTGGAATTCATCATTTTCAATGTCTTGCCCTTAGAATGArGrU |
| CDCP1（173）Probe-L2 | PO_4_CTTGCCCTTAGAATGAGTCCACTGAGCAATGTGAAGTTGGCTCTATGGGCAGTCGGTGAT |
| CDCP1（173）Probe-R2 | CCATCTCATCCCTGCGTGTCTCATGACTGTATCCAGATTGGAATTCATCATTTTCAATrGrU |
| CDCP1（212）Probe-L3 | PO_4_CATGACTGTATCCAGATTGGAATTCATCATTTTCAATGTCCTCTATGGGCAGTCGGTGAT |
| CDCP1（212）Probe-R3 | CCATCTCATCCCTGCGTGTCGGCTAACCGCACAGCCTAAGTTGAGGAGCACATGAGCTrGrU |
| CDCP1control ProbeL | PO_4_ATCCAGATTGGAATTCATCATTTTCAATGTCTTGCCCTTACTCTATGGGCAGTCGGTGAT |
| CDCP1control ProbeR | CCATCTCATCCCTGCGTGTCCACAGCCTAAGTTGAGGAGCACATGAGCTGTCATGACTrGrU |
| m^6^A universal primer -F | ATCACCGACTGCCCATAGAG |
| m^6^A universal primer -R | CGCACAGCCTAAGTTGAGGA |

**Supplementary Table 4.** RT-qPCR primer sequences

| CDCP1 3'UTR-F | CCGCCAACTTCACATTGCTC |
| --- | --- |
| CDCP1 3'UTR-R | CGCACAGCCTAAGTTGAGGA |
| EGFR-F | GTCGGGCTCTGGAGGAAAA |
| EGFR-R | ATTCCCAAGGACCACCTCAC |
| OR4R5-F | GGAGTGTCCGTCCTCATGTA |
| OR4R5-R | TGGGATCCTTGACTTGCCC |
| AP4S1-F | GCCTTTGATACTCATTTTAAGGGCA |
| AP4S1-R | CTGGGGCTGTCTTAATGTCTGA |
| SPECC1L -F | CCAGCTGTCTGGTCATGGTT |
| SPECC1L -R | GAACTGCAGACCAGGGGAAG |
| SLC22A9-F | TCCCTCTTTGAACCTCTCTGGA |
| SLC22A9-R | GTCACCAGCGTGACCCAG |
| ST18-F | TCAACTCCACGTCGAACTCAA |
| ST18-R | AAAGGAATGGTGAGGGGTGG |
| ROR1-F | CCCAGAAGCTGCGAACTGTA |
| ROR1-R | GTAGTCCACACCTGTGCTGT |

**Supplementary Sequences 1.** Amino acid sequences of nucleus-localized RCas9-M3 editors. The color coding is as follows:

grey = Kozak sequence

orange = METTL3^357-580^ (for dCas9–M3)

black = linkers

blue = Cas9 ΔD10A, H840A (dCas9)

yellow = HA

red = SV40 nuclear localization signal

green = EGFP

ATMQSVGGDSSADRLFPPQWICCDIRYLDVSILGKFAVVMADPPWDIHMELPYGTLTDDEMRRLNIPVLQDDGFLFLWVTGRAMELGRECLNLWGYERVDEIIWVKTNQLQRIIRTGRTGHWLNHGKEHCLVGVKGNPQGFNQGLDCDVIVAEVRSTSHKPDEIYGMIERLSPGTRKIELFGRPHNVQPNWITLGNQLDGIHLLDPDVVARFKQRYPDGIISKPKNL**GGSGGGSMDKKYSIGLAIGTNSVGWAVITDEYKVPSKKFKVLGNTDRHSIKKNLIGALLFDSGETAEATRLKRTARRRYTRRKNRICYLQEIFSNEMAKVDDSFFHRLEESFLVEEDKKHERHPIFGNIVDEVAYHEKYPTIYHLRKKLVDSTDKADLRLIYLALAHMIKFRGHFLIEGDLNPDNSDVDKLFIQLVQTYNQLFEENPINASGVDAKAILSARLSKSRRLENLIAQLPGEKKNGLFGNLIALSLGLTPNFKSNFDLAEDAKLQLSKDTYDDDLDNLLAQIGDQYADLFLAAKNLSDAILLSDILRVNTEITKAPLSASMIKRYDEHHQDLTLLKALVRQQLPEKYKEIFFDQSKNGYAGYIDGGASQEEFYKFIKPILEKMDGTEELLVKLNREDLLRKQRTFDNGSIPHQIHLGELHAILRRQEDFYPFLKDNREKIEKILTFRIPYYVGPLARGNSRFAWMTRKSEETITPWNFEEVVDKGASAQSFIERMTNFDKNLPNEKVLPKHSLLYEYFTVYNELTKVKYVTEGMRKPAFLSGEQKKAIVDLLFKTNRKVTVKQLKEDYFKKIECFDSVEISGVEDRFNASLGTYHDLLKIIKDKDFLDNEENEDILEDIVLTLTLFEDREMIEERLKTYAHLFDDKVMKQLKRRRYTGWGRLSRKLINGIRDKQSGKTILDFLKSDGFANRNFMQLIHDDSLTFKEDIQKAQVSGQGDSLHEHIANLAGSPAIKKGILQTVKVVDELVKVMGRHKPENIVIEMARENQTTQKGQKNSRERMKRIEEGIKELGSQILKEHPVENTQLQNEKLYLYYLQNGRDMYVDQELDINRLSDYDVDAIVPQSFLKDDSIDNKVLTRSDKNRGKSDNVPSEEVVKKMKNYWRQLLNAKLITQRKFDNLTKAERGGLSELDKAGFIKRQLVETRQITKHVAQILDSRMNTKYDENDKLIREVKVITLKSKLVSDFRKDFQFYKVREINNYHHAHDAYLNAVVGTALIKKYPKLESEFVYGDYKVYDVRKMIAKSEQEIGKATAKYFFYSNIMNFFKTEITLANGEIRKRPLIETNGETGEIVWDKGRDFATVRKVLSMPQVNIVKKTEVQTGGFSKESILPKRNSDKLIARKKDWDPKKYGGFDSPTVAYSVLVVAKVEKGKSKKLKSVKELLGITIMERSSFEKNPIDFLEAKGYKEVKKDLIIKLPKYSLFELENGRKRMLASAGELQKGNELALPSKYVNFLYLASHYEKLKGSPEDNEQKQLFVEQHKHYLDEIIEQISEFSKRVILADANLDKVLSAYNKHRDKPIREQAENIIHLFTLTNLGAPAAFKYFDTTIDRKRYTSTKEVLDATLIHQSITGLYETRIDLSQLGGDAYPYDVPDYASLGSGS****PKKKRKVEDPKKKRKVDGIGSGSNGSSGSMVSKGEELFTGVVPILVELDGDVNGHKFSVSGEGEGDATYGKLTLKFICTTGKLPVPWPTLVTTLTYGVQCFSRYPDHMKQHDFFKSAMPEGYVQERTIFFKDDGNYKTRAEVKFEGDTLVNRIELKGIDFKEDGNILGHKLEYNYNSHNVYIMADKQKNGIKVNFKIRHNIEDGSVQLADHYQQNTPIGDGPVLLPDNHYLSTQSALSKDPNEKRDHMVLLEFVTAAGITLGMDELYK**

**Supplementary Sequences 2.** Sequences of hU6 promoter-2 × BbsI-sgRNA scaffold constructs. The color coding is as follows:

grey = human U6 promoter

green = BbsI restriction enzyme sites

yellow = sgRNA scaffold

GAGGGCCTATTTCCCATGATTCCTTCATATTTGCATATACGATACAAGGCTGTTAGAGAGATAATTAGAATTAATTTGACTGTAAACACAAAGATATTAGTACAAAATACGTGACGTAGAAAGTAATAATTTCTTGGGTAGTTTGCAGTTTTAAAATTATGTTTTAAAATGGACTATCATATGCTTACCGTAACTTGAAAGTATTTCGATTTCTTGGCTTTATATATCTTGTGGAAAGGACGAAACACCGG***GTCTTC***GA***GAAGAC***CTGTTTAAGAGCTATGCTGGAAACAGCATAGCAAGTTTAAATAAGGCTAGTCCGTTATCAACTTGAAAAAGTGGCACCGAGTCGGTGCTTTTTTT

**Supplementary Sequences 3.** Sequences of 2xBsmBI-sgRNA scaffold-cppt/cts constructs used to construct the lentiviral vector. The color coding is as follows:

green = BsmBI restriction enzyme sites

yellow = sgRNA scaffold

purple = cPPT/cts

CACCG***GAGACG***GA***CGTCTC***TGTTTAAGAGCTATGCTGGAAACAGCATAGCAAGTTTAAATAAGGCTAGTCCGTTATCAACTTGAAAAAGTGGCACCGAGTCGGTGCTTTTTTAAGCTTGGCGTAACTAGATCTTGAGACAAATGGCAGTATTCATCCACAATTTTAAAAGAAAAGGGGGGATTGGGGGGTACAGTGCAGGGGAAAGAATAGTAGACATAATAGCAACAGACATACAAACTAAAGAATTACAAAAACAAATTACAAAAATTCAAAATTTTCGGGTTTATTACAGGGACAGCAGAGATCCACTTTGGCGCCGGC

**Methods**

**Plasmid construction, PAMmer synthesis, and target site choice**

The METTL3 catalytic domain (METTL3CD) and dCas9-2xNLS sequences were amplified from HUVEC and pHR-SFFV-KRAB-dCas9-P2A-mCherry plasmids (a gift from Jonathan Weissman; Addgene #60954), respectively. The EGFP sequence was amplified from a pLKO.3G plasmid (a gift from Christophe Benoist & Diane Mathis; Addgene #14748). The original pHR-SFFV-KRAB-dCas9-P2A-mCherry vector was sequentially digested with NotI and MluI(New England Biolabs, USA), and the vector pCDNA 3.1 (Life Technologies, CA, USA) was digested with the restriction endonucleases BamHI and xhoI(New England Biolabs, USA). METTL3CD, dCas9-2xNLS, and EGFP were incorporated into the two vectors using Gibson assembly to generate 60954-Mettl3 and pcDNA-Mettl3 plasmids. In addition, a dCas9-EGFP fusion protein was used as a negative control. sgRNA scaffolds were constructed as described [1]. The sgRNA scaffold containing the human U6 polymerase III promoter and two BsmBI restriction sites at the 5’ end was synthesized and cloned into the multiple cloning site of pBlueScript II SK (+) (Agilent) and Lentiguide-puro (a gift from Feng Zhang; Addgene #52963) by Gibson assembly. The sgRNA scaffold constructs were digested with BsmBI (New England Biolabs, USA) to produce sgRNAs targeting the 3’UTR of *CDCP1* and epidermal growth factor receptor (*EGFR*) mRNAs. Primer sequences for PCR amplification are summarized in Supplementary Table 1.

We chose RCas9 target sites using the IDT antisense oligonucleotide design tool, and the microarray probe design tools Picky[2]and OligoWiz[3]. Then, we designed PAMmers against high-confidence sites and included eight bases on the 5’ end beyond the PAM sequence. The PAMmers were composed of mixed 2’ OMe RNA and DNA bases and were purified by HPLC [1]. The sgRNAs and PAMers used in this study are listed in Supplementary Table 2.

**Cell lines**

Human uroepithelial cells (SV-HUC-1), HeLa cells and 293T cells were obtained from the American Type Culture Collection (Manassas, VA). BC cell line T24 was purchased from the Institute of Cell Biology, Chinese Academy of Sciences (Shanghai, China). BC cell line T24 cells were grown in RPMI 1640. HeLa cells and 293T cells were cultured in DMEM, and SV-HUC-1 cells were maintained in F-12K (Fisher Scientific, MA, USA). All media were supplemented with 10% fetal bovine serum (Gibco, USA) and penicillin/streptomycin (Life Technologies, CA, USA). Cells were maintained in a humidified incubator at 37 °C with 5% CO_2_.

**Lentiviral transduction to establish stable cell lines**

For virus transduction, 293T cells were transfected with the appropriate lentiviral vector using the Lipofectamine 3000 reagent (Invitrogen, MA, USA). Lentiviral vectors expressing METTL3CD-dCas9-2XNLS-EGFP, dCas9-2XNLS-EGFP, and sgRNAs targeting *CDCP1* or *EGFR* were generated as described above. The target plasmid and the packaging vectors psPAX2(a gift from Didier Trono; Addgene plasmid # 12260) and VSVG (a gift from Bob Weinberg; Addgene plasmid # 8454) were co-transfected into 293T cells at a ratio of 1:1:0.5. To establish stable cell lines, target cells were transduced using the above lentiviruses with 8 μg /mL polybrene (Sigma, Germany). Seventy-two hours after transduction, cells were selected with 2 μg/mL puromycin for 5-7 days.

***CDCP1* mRNA targeting with RCas9-METTL3**

The stable cells were cultured as described above till they reached 80% confluency. Next, PAMmers were transfected using Lipofectamine RNAiMax (Life Technologies, CA, USA). Twenty-four hours after transfection, cells were washed with phosphate-buffered saline (PBS), fixed with 4% paraformaldehyde for 15 min, and washed again with PBS. Nuclei were counterstained with DAPI (Life Technologies, CA, USA). Images were taken with a Zeiss LSM 880 confocal microscope.

**m^6^A methylated RNA immunoprecipitation (MeRIP)**

Cells stably expressing RCas9-M3 cultured as described above and were passaged at 80% confluency. PAMmers were transfected using Lipofectamine RNAiMax (Life Technologies) according to manufacturer’s instructions. Total RNA was extracted from cells 24 h after transfection using TRIzol (Invitrogen, MA, USA) and components for targeted methylation of *CDCP1* or *EGFR* 3′ UTR. mRNA was purified from total RNA using a Gen Elute mRNA Miniprep Kit (Sigma-Aldrich, Germany). Next, 10 μg mRNA was fragmented into 200-300 nt long fragments using a RNA fragmentation kit (Ambion, USA) and immunoprecipitated with protein A/G beads (Thermo Fisher, MA, USA) coated with anti-m^6^A antibody (Synaptic Systems, #202003). After immunoprecipitation, RNA was washed and eluted from the beads with m^6^A elution buffer (Sigma-Aldrich) and the purified RNA fragments were used for RT- qPCR analysis.

**Single-base validation using a ligase-based method**

SV-HUC-1 cells stably expressing RCas9-M3 were generated using the lentivirus constructed above and following the single-based T3 ligase-based method[4, 5]. DNA probes L and R were designed to match the flanking sequences of intended site. Probe L (left) was modified with an additional phosphate group at the 5′ end. Probe R (right) was designed with two modified ribonucleotides at the 3′ end (see Supplementary Table 3). The ligation reaction was performed in an 8 μL volume containing 300 ng total RNA, 20 nM probe L, 20 nM probe R, and 1× T3 ligation buffer (New England Biolabs, USA).Then, the reaction mixture was incubated at 85 °C for 3 min and at 35 °C for 10 min. 50 U T3 DNA ligase and 1×ligation buffer were added to bring the final volume to 10 μL. The mixture was incubated at 35 °C for 10 minutes and then placed on ice. For PCR amplification, 1 μL ligation product was amplified by PCR with 2× Taq mix (Vazyme Biotech, Nanjing, China) for 35 cycles, using the following cycles: 94°C for 2 min., 94°C for 45 S., 58°C for 40 S., and 72°C for 40 S. All PCR products were analyzed by 2% agarose gel electrophoresis using 1× tris-acetate-EDTA (TAE) buffer.

**Dual-luciferase reporter assay**

The 3’ UTR of *CDCP1* was cloned from HUVEC cells into psiCHECK-2 vector (Promega, USA) to generate psiCHECK-2-CDCP1-3’ UTR. 293T and SV-HUC-1 cells cultured as described above and were passaged at 80% confluency. A total of 300,000 cells were seeded into each well of 24-well tissue culture plates. After 24 hours, cells were co-transfected with either dCas9-M3 or sgRNA and psiCHECK-2-CDCP1-3’ UTR Fluc reporter plasmid at a mass ratio of 5:3:1 using Lipofectamine 3000 (Life Technologies CA, USA). PAMmers were transfected using Lipofectamine RNAiMax (Life Technologies) according to manufacturer’s instructions. Forty-eight hours after transfection, cells were washed with PBS and lysed with Reporter Lysis Buffer (Promega, USA). The relative luci­ferase activity of the lysates was measured with the Dual-Glo Luciferase Assay System (Promega, USA) on a SYNERGY microplate reader (BioTek, USA).

**Immunoblotting (Western blot)**

Cells were washed twice with ice-cold PBS and lysed with RIPA buffer (Sigma-Aldrich, Germany) containing protease inhibitor cocktail. The solution was centrifuged for 20 min at 10,000 × g and the supernatant was collected. Cell lysates were separated by SDS-PAGE and transferred to PVDF membranes. Membranes were blocked with 5% BSA in Tris-buffered saline (pH 7.5) containing 0.1% Tween 20 for 1 hour and incubated overnight at 4 °C with the following primary antibodies: anti-rabbit METTL3 antibody (1:1,000, Proteintech,15073-1-AP), anti-rabbit *CDCP1* antibody (1:1,000, Cell Signal Technology, 4115), anti-mouse *EGFR* antibody (1:1,000, Proteintech,66455-1-Ig), anti-rabbit β-Actin antibody (1:1,000, Cell Signal Technology, 4967), and anti-mouse GFP antibody (1:1,000, Santa Cruz, #K0217). Next, membranes were washed with Tris-buffered saline with 0.1% Tween 20 and incubated with Anti-rabbit IgG HRP-linked(1:5,000, Cell Signal Technology, 7074) or Anti-mouse IgG HRP-linked (1:5,000, Cell Signal Technology, 7076) secondary antibodies at room temperature for 1 h. ECL Western Blotting Substrate (Thermo Fisher, USA) was used for detection.

**Immunostaining**

The cells were fixed with 4% paraformaldehyde in PBS for 20 min, blocked with 1% BSA (bovine serum albumin) for 1 h at room temperature, and incubated for 2 h at room temperature with anti-rabbit *CDCP1* antibody (1:1,00, Cell Signal Technology, 4115)primary antibodies diluted in block­ing solution. Next, the cells were incubated with Alexa Fluor 568 donkey anti-rabblit IgG(H+L) (1:1,000, Invitrogen, 1891789) for 1 h at room temperature.1× DAPI (Solarbio, S2110) was used for nuclear staining and the images were taken with a confocal microscope (LSM880, Carl Zeiss).

**RNA isolation and quantitative real-time PCR**

RNA was purified using TRIzol (Life Technologies) and the cDNA was synthesized using TransScript All-in-One First-Strand cDNA Synthesis SuperMix for qPCR (Vazyme Biotech, Nanjing, China). qPCR was performed using Fast SYBR Green PCR Master Mix (Applied Biosystems, USA) on a Step-One Fast Real-time PCR System (Applied Biosystems, USA). The primers used for qPCR are listed in Supplementary Table 4.

**RNA-seq analysis**

Total RNA was isolated using TRIzol reagent. The RNA concentration and quality were measured with a NanoDrop 2000 (Thermo Fisher). RNA integrity and gDNA contamination were evaluated using denaturing agarose gel electrophoresis. Purified RNA was further purified with a RiboMinus Eukaryote Kit (Qiagen) to remove ribosomal RNA prior to RNA-seq library construction. Sequencing library concentration was determined with an Agilent 2100 Bioanalyzer using a DNA 1000 chip kit (Agilent, CA, USA). The libraries were adjusted to 10 nM before cluster generation. Finally, the samples were analyzed by 100 bp paired-end sequencing using a HiSeq 2000 system (Illumina, San Diego, CA, USA). RNA-seq analysis Read counts were imported into Rstudio (version 1.0.136) and normalized across samples using the ‘DESeq2’ package, which was also used for conducting the differential gene expression analysis.

**RNA Immunoprecipitation**

Stable cells containing the RCas9-M3 were cultured as described above were passaged at 80% confluency. Next, cells were transfected with PAMmers using Lipofectamine RNAiMax (Life Technologies). After 48 hours, the cell lysate was immunoprecipitated using a Magna RIP RNA-Binding Protein Immunoprecipitation Kit (Millipore, MA, USA), 5 μg anti-EGFP antibody (Santa Cruz, K0217), and 50 μL protein A/G magnetic beads at room temperature for 30 minutes. The magnetic beads were immobilized with a magnet and washed with RIP Wash Buffer (Millipore, MA, USA). The precipitated RNA was extracted with phenol: chloroform, and the relative abundance of CDCP1 RNA on the beads was compared to that in supernatant by qPCR.

**Proliferation assay**

Stable cells(3000/well) containing the RCas9-M3 were seeded into 96-well plates and incubated at 37 °C and 5% CO_2_ in a humidified incubator. Cell proliferation was quantified using a Cell Titer 96 AQueous One Solution Cell Proliferation Assay (MTS) kit (Promega, USA). Briefly, 20 μL MTS solution was added to each well after 24, 48, 72, and 96 h and incubated at 37 °C for 2 hours. The absorbance at 490 nm was determined using a Synergy microplate reader (BioTEK, USA).

**Migration assay**

Cell migration assay was performed using an IncuCyte 96-Well Real-Time Cell Migration System (Essen Bioscience, USA). Cells (1 × 10^5^/well) were plated in a 96-well Essen ImageLock plates(Essen Bioscience, USA) and grown to confluence in standard a CO_2_ incubator. A 96-pin WoundMaker was used to create precise and reproducible wounds through the confluent cell monolayer. The plate was washed with cold PBS and scanned every hour for 24 h. The data were analyzed using the Relative Wound Density and images were acquired using phase contrast imaging. Data were analyzed with GraphPad Prism software.

**Cell invasion assay**

Transwell Matrigel invasion assays were performed using 24-well transwell inserts with an 8-μm pore size (Corning). First, a 24-well permeable support plate was coated with 200–300 μg/mL Corning Matrigel matrix (Corning Cat. No. 354234). After 24 h, 1 × 10^5^ cells in 200 μL culture medium without FBS were plated in the upper chambers and 500 μL culture medium containing 20% FBS was placed in the lower chambers. The plates were incubated for 24 h at 37 °C. The invading cells were fixed with 100% methanol for 30 min and stained with 0.1% crystal violet for 30 min at room temperature. The invading cells were counted using a Zeiss Axio Imager.Z2 microscope.

**Tumorigenicity in NOD/SCID mice**

All animal experimental procedures were approved by the Institutional Ethics Committee for Clinical Research and Animal Trials of the First Affiliated Hospital of Sun Yat-sen University. For the subcutaneous implantation, 5 × 10^6^ cells were subcutaneously implanted into 5-week-old BALB/cJNju-Foxn1nu/Nju nude mice (Nanjing Biomedical Research Institute of Nanjing University). Tumor formation and growth were assessed weekly and the mice were sacrificed 4 weeks after surgery. Tumor volume was calculated as:

Tumor volume=Length×Width2×0.52.

**Statistical analysis**

Statistical analysis was performed with the GraphPad Prism 8.0 (GraphPad, La Jolla, CA, USA) and SPSS 22.0 (SPSS, La Jolla, CA, USA) software. Experiments were independently repeated for at least three times. The results are presented as the mean ± SEM. was analyzed by unpaired Student’s t test or one-way ANOVA with Dunnett’s multiple comparisons using GraphPad Prism. P-values for every result were labeled on figures, and P < 0.05 was reckoned as statistically significant (*P < 0.05, **P < 0.01, ***P < 0.001, ****P < 0.0001).

**REFERENCES**

1 Nelles DA, Fang MY, O'Connell MR, Xu JL, Markmiller SJ, Doudna JA, Yeo GW. Programmable RNA Tracking in Live Cells with CRISPR/Cas9. Cell. 2016;165:488-96.

2 Chou HH, Hsia AP, Mooney DL, Schnable PS. Picky: oligo microarray design for large genomes. Bioinformatics. 2004;20:2893-902.

3 Wernersson R, Nielsen HB. OligoWiz 2.0--integrating sequence feature annotation into the design of microarray probes. Nucleic Acids Res. 2005;33:W611-5.

4 Liu W, Yan J, Zhang Z, Pian H, Liu C, Li Z. Identification of a selective DNA ligase for accurate recognition and ultrasensitive quantification of N(6)-methyladenosine in RNA at one-nucleotide resolution. Chem Sci. 2018;9:3354-59.

5 Zhang Z, Chen LQ, Zhao YL, Yang CG, Roundtree IA, Zhang Z, Ren J, Xie W, He C, Luo GZ. Single-base mapping of m(6)A by an antibody-independent method. Sci Adv. 2019;5:eaax0250.
